# Effect of forefoot transverse arch stiffness on foot biomechanical response--based on finite element method

**DOI:** 10.3389/fbioe.2024.1387768

**Published:** 2024-07-08

**Authors:** Linjie Zhang, Qiaolin Zhang, Yilin Zhong, Tibor Hortobagyi, Yaodong Gu

**Affiliations:** ^1^ Department of Radiology, Ningbo No. 2 Hospital, Ningbo, China; ^2^ Department of Kinesiology, Hungarian University of Sports Science, Budapest, Hungary; ^3^ Doctoral School of Safety and Security Sciences, Obuda University, Budapest, Hungary; ^4^ Faculty of Engineering, University of Szeged, Szeged, Hungary; ^5^ Faculty of Sport Science, Ningbo University, Ningbo, China

**Keywords:** forefoot transverse arch, foot stiffness, stress distribution, metatarsal stress, finite element model, plantar fascia strain

## Abstract

**Background:**

The plantar vault, comprising the transverse and longitudinal arches of the human foot, is essential for impact absorption, elastic energy storage, and propulsion. Recent research underscores the importance of the transverse arch, contributing over 40% to midfoot stiffness. This study aimed to quantify biomechanical responses in the ankle-foot complex by varying the stiffness of the deep metatarsal transverse ligament (DTML).

**Methods:**

Using CT image reconstruction, we constructed a complex three-dimensional finite element model of the foot and ankle joint complex, accounting for geometric complexity and nonlinear characteristics. The focus of our study was to evaluate the effect of different forefoot transverse arch stiffness, that is, different Young’s modulus values of DTML (from 135 MPa to 405 MPa), on different biomechanical aspects of the foot and ankle complex. Notably, we analyzed their effects on plantar pressure distribution, metatarsal stress patterns, navicular subsidence, and plantar fascial strain.

**Results:**

Increasing the stiffness of the DTML has significant effects on foot biomechanics. Specifically, higher DTML stiffness leads to elevate von Mises stress in the 1st, 2nd, and 3rd metatarsals, while concurrently reducing plantar pressure by 14.2% when the Young’s modulus is doubled. This stiffening also impedes navicular bone subsidence and foot lengthening. Notably, a 100% increase in the Young’s modulus of DTML results in a 54.1% decrease in scaphoid subsidence and a 2.5% decrease in foot lengthening, which collectively contribute to a 33.1% enhancement in foot longitudinal stiffness. Additionally, doubling the Young’s modulus of DTML can reduce the strain stretch of the plantar fascia by 38.5%.

**Conclusion:**

Preserving DTML integrity sustains the transverse arch, enhancing foot longitudinal stiffness and elastic responsiveness. These findings have implications for treating arch dysfunction and provide insights for shoe developers seeking to enhance propulsion.

## 1 Introduction

In various human physical activities including walking, running, jumping, and other sports, the foot serves as the terminal point of movement. It functions to attenuate the impact forces from ground contact during landing (Chan and Rudins, 1994; Pan et al., 2023), generate propulsive force for the body during push-off (Bramble and Lieberman, 2004; Takahashi et al., 2016; Xu et al., 2024), and optimize energy conversion efficiency (Kuo et al., 2005; Zelik and Kuo, 2010; Khuyagbaatar et al., 2024). To accommodate the multifunctional demands placed upon them, humans have evolved a pair of remarkably flexible feet capable of modulating stiffness to suit various requirements across different athletic endeavors (Bojsen-Møller, 1979; Ker et al., 1987; Kuo et al., 2005; Zelik and Kuo, 2010). The differential stiffness observed in the foot can largely be attributed to the structural composition of the plantar vault, which is formed by the medial longitudinal arch (MLA), lateral longitudinal arch (LLA), and transverse arch (TA). These arches play a significant role in determining the overall stiffness characteristics of the foot (Gwani et al., 2017; Venkadesan et al., 2020).

The MLA has traditionally been a focal point for researchers investigating foot elasticity and mechanical functionality (Morton, 1924b; Elftman and Manter, 1935; Hicks, 1954a; Bojsen-Møller, 1979; Susman, 1983; Williams and McClay, 2000; Heard-Booth, 2017; Holowka and Lieberman, 2018). It is widely recognized as a primary determinant of the foot’s elastic response in the sagittal plane and contributes significantly to midfoot stiffness. Working in conjunction with the bow-string configuration established by the plantar fascia (PF) (Morton, 1924a; Ker et al., 1987) and the windlass mechanism facilitated by dorsiflexion of the metatarsophalangeal joint (Hicks, 1954b), the MLA contributes to midfoot lengthening and stiffness. During forefoot loading, ground reaction forces compel passive stretching of the PF (Morton, 1924b; Ker et al., 1987). Similarly, dorsiflexion of the metatarsophalangeal joints during foot propulsion also results in stretching of the PF (Hicks, 1954b). These two mechanisms of stretching the PF serve to impede MLA collapse and are directly correlated with MLA height (Williams and McClay, 2000). Furthermore, a cadaveric investigation revealed a reduction in foot stiffness following transection of the PF, albeit this reduction was limited to less than 25% (Huang et al., 1993). In light of these observations, the hypothesis proposing that MLA height correlates with foot stiffness emerged, leading to the arch height index becoming a widely utilized metric for foot stiffness assessment (Williams and McClay, 2000; Xiang et al., 2024). However, the premise of utilizing MLA height as a proxy for adequate stiffness possesses inherent limitations, as evidenced by several studies. For instance, individuals with MLA collapse can exhibit normal gait patterns (DeSilva et al., 2015). Even in cases where the PF is transversely severed, foot stiffness diminishes by only a fraction, as indicated in previous research (Huang et al., 1993). In a comparative analysis of individuals with normal arches and flat feet, Kido et al. (2013) observed that midfoot deformation under body weight loading was twice as pronounced in patients with flat feet, with the disparity in stiffness between normal arches and flat feet surpassing the contribution of the PF. Moreover, in conditions characterized by low MLA height such as diabetic foot and peripheral neuropathy, the winch mechanism persists but fails to furnish adequate stiffness support (Gelber et al., 2014). Collectively, these findings underscore the inadequacy of solely relying on sagittal plane foot mechanics to elucidate foot stiffness, signaling the need for a deeper understanding of foot biomechanics.

The TA comprises two bony structures exhibiting slight curvature in the vertical direction of the MLA, situated at the tarsometatarsal joint and metatarsophalangeal joint, respectively (Ridola and Palma, 2001). Biomechanical investigations of the TA have been relatively scarce in recent years, with the majority focusing on the forefoot transverse arch (FTA) at the metatarsophalangeal joint (Iaremenko, 1967; Daentzer et al., 1997; Luger et al., 1999; Weishaupt et al., 2002; Kanatli et al., 2003; Masłoń et al., 2017; Nakai et al., 2019). Plantar pressure data appear to contradict the anatomical configuration and function of the FTA during foot loading (Daentzer et al., 1997; Kanatli et al., 2003), as higher plantar pressure is observed beneath the 2nd and 3rd metatarsals compared to other metatarsals. Conversely, Powell et al. (2014) conducted X-ray imaging and measurements of the forefoot under maximal load in 200 randomly selected Danish subjects, affirming the persistent presence of a bony FTA in the forefoot with an average height of 1.4 mm even under maximum load. Recent investigations have underscored the significance of the TA in contributing to midfoot stiffness, thereby addressing the inadequacy of utilizing MLA height as a sole indicator of foot stiffness (Venkadesan et al., 2020). Venkadesan et al. (2020) demonstrated through mechanical simulations that the TA constitutes the primary determinant of foot stiffness, accounting for over 40% of total foot stiffness. This phenomenon stems from the mechanical coupling between sagittal plane bending of the foot and perpendicular stretching of the metatarsal heads, akin to the significant stiffening observed when folding a banknote crosswise. Yawar et al. (2017) conducted experiments involving subjects with FTA wrapped in elastic bandages and employed mathematical models to ascertain that augmenting the lateral stiffness of the FTA resulted in an average increase of 53% in foot stiffness. Furthermore, they posited that the orientation of the adjacent metatarsal joint axis carried more significance than the external curvature in influencing foot biomechanics. Despite the absence of an overt transverse arch in some foot configurations, the geometric features of the tarsal/metatarsal joints and ligament arrangement may lead to misalignment of the preferred bending direction of adjacent metatarsals. This functional bending capability enables the storage of elastic potential energy generated by the stretching of interosseous ligaments. Further exploration is warranted to elucidate the relationship between the TA and foot stiffness. Schmidt et al. (2024) conducted a retrospective analysis of weight-bearing CT images from 32 Progressive collapsing foot deformity and 32 control feet, revealing a greater degree of TA collapse in progressively collapsing clubfeet compared to controls. The most significant collapse was observed between the medial cuneiform and the second metatarsal bones. This observation suggests a potential coupling mechanism between the TA and the MLA, specifically occurring between the medial cuneiform and the second metatarsal. Moreover, the biomechanical responses of different FTA lateral stiffnesses on foot mechanics remain unexplored and merit investigation.

In 1973, Belytschko et al. (1973) pioneered the application of the finite element (FE) method in biomechanical research. This method has gained widespread popularity due to its capacity to conduct iterative mechanical analyses of structures characterized by irregular geometric shapes and intricate material properties within complex boundaries. It stands as one of the foremost methodologies in foot biomechanics research (Yu et al., 2020). Consequently, we aimed to construct a three-dimensional FE model of the foot-ankle complex using CT data obtained from healthy subject. The lateral stiffness of the FTA within the foot-ankle complex was manipulated by adjusting the Young’s modulus of the DTML. Comprising a series of four short ligaments spanning the distal ends of adjacent metatarsals, the DTML plays a pivotal role in stabilizing deformations of the foot’s transverse arch (Wang et al., 2015). Our objective is to investigate the corresponding impacts of varying FTA lateral stiffness on von Mises stress, strain, and plantar pressure across foot bones and PF tissue under identical loading conditions.

We hypothesized that augmenting the lateral stiffness of the FTA would mitigate forefoot plantar pressure, induce alterations in metatarsal von Mises stress and stress distribution, diminish navicular bone descent, and attenuate PF strain.

## 2 Materials and methods

### 2.1 Participant information

This study included one healthy male subject (age: 26 years, height: 186 cm, weight: 75 kg). The subject’s feet exhibited no signs of neurological disease, biomechanical abnormalities resulting from acute foot injuries, or previous foot bone surgeries, and there were no hereditary foot deformities observed. Prior to measurements, subjects were provided with comprehensive information regarding the experimental procedures and were required to sign an informed consent form. Ethical approval for this study was obtained from the Human Subjects Ethics Committee of Ningbo University (RAGH20230428), and all laboratory procedures adhered to the principles outlined in the Declaration of Helsinki.

### 2.2 Model construction

**FIGURE 1:**
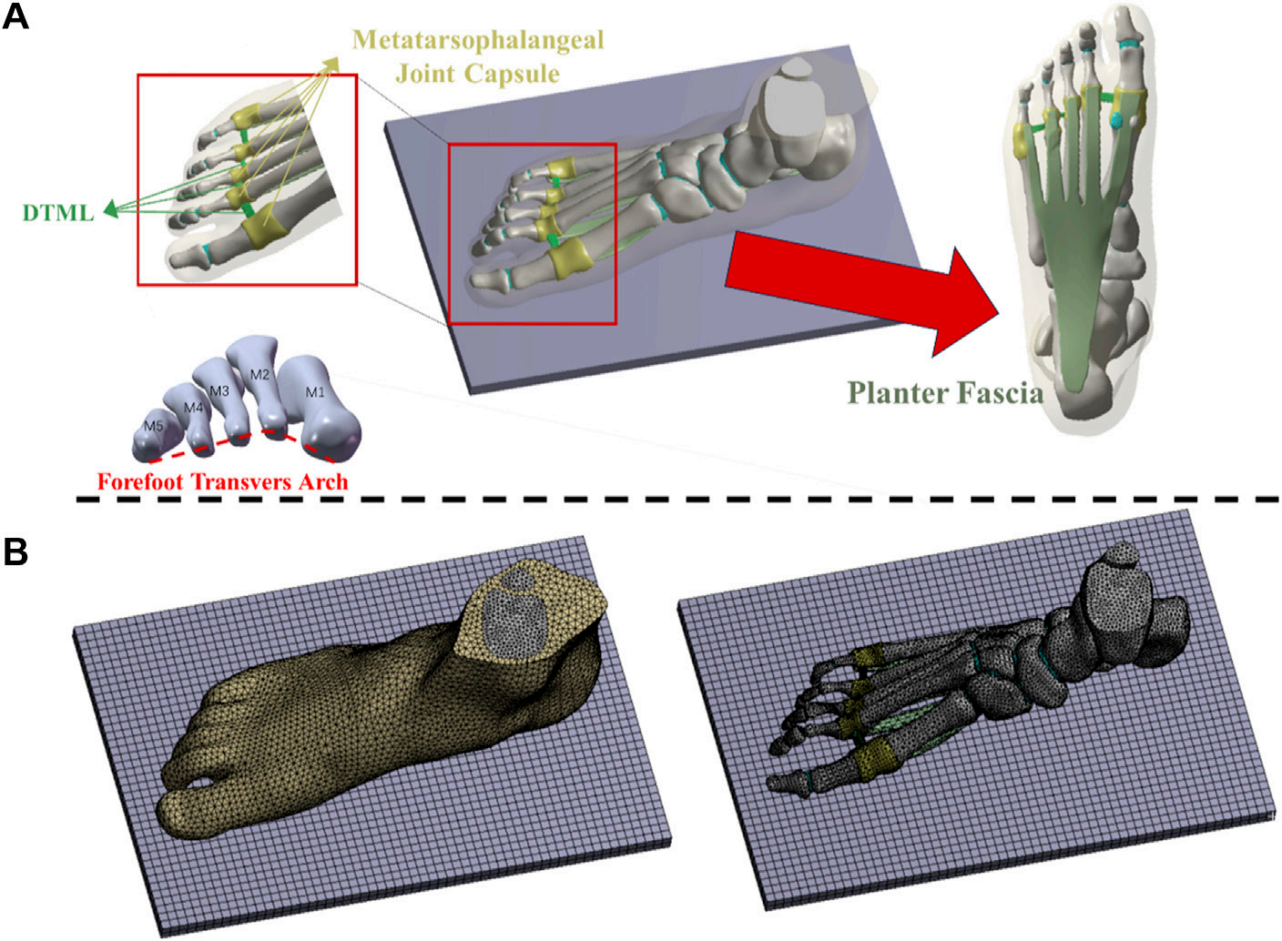
**(A)** Three-dimensional finite element models of soft tissue, bone and ligament structures; **(B)** Mesh model of foot-ankle complex under load.

A coronal CT scan, conducted without weight bearing, was performed on the subject’s right foot in a neutral position, with a 2 mm interval between slices. The DICOM image was segmented using Mimics16.0 (Materialise, Leuven, Belgium) to generate a three-dimensional model encompassing both bone tissue and capsular soft tissue. Geometric irregularities present on the surfaces of bony components and soft tissues were smoothed using Geomagic Studio 2013 (Geomagic Inc, Research Triangle Park, North Carolina, United States). Subsequently, a PF model was established based on foot anatomy (Tao et al., 2010). Each surface member was individually imported into SolidWorks 2016 (Massachusetts, United States, SolidWorks) to create solid parts. A solid cartilage structure was constructed based on the bone contact surface. Volumetric Boolean operations were performed to subtract all bone and cartilage components, resulting in the encapsulated soft tissue being derived from the total soft tissue. The numerical foot model comprises 28 bone segments, which include the tibia, fibula, talus, calcaneus, cuboid, navicular, 3 cuneiforms, 5 metatarsals, and 14 phalanges (Zhang et al., 2022) (refer to Figure 1A).

### 2.3 Geometry reconstruction and mesh creation

The mesh was generated utilizing the Ansys Workbench (ANSYS, Inc., United States) grid tool, as illustrated in Figure 1B. Each bone, cartilage component, and surrounding soft tissue was segmented into sub volumes, with tetrahedral elements assigned to each volume. The mesh sizes for the two models were set as follows: 4.5 mm for the encapsulated soft tissue, 3 mm for the bone, and 2.5 mm for the cartilage structure. Local refinement was implemented to accommodate contact areas and fine geometries. The solid part was meshed using tetrahedral elements. Except for the PF, DTML, and metatarsophalangeal joint capsule, other ligaments are non-solid line units that only stretch. Employing a method of gradually reducing mesh size, a mesh sensitivity test was conducted on the full-foot model at a static station. Acceptable mesh generation was determined by evaluating the deviation of peak equivalent von Mises stress synthesized by the first metatarsal bone. Further mesh reduction was undertaken until the von Mises stress deviation remained within 5% of the original value (Chen et al., 2019).

### 2.4 Material property assignment

With the exception of the encapsulated soft tissue, all materials are modeled as isotropic and linearly elastic (Cheung et al., 2005). Two material constants, Young’s modulus (E) and Poisson’s ratio (ν), are defined to characterize elasticity. The soft tissue is treated as a nonlinear hyper elastic material. The material properties of the soft tissue are derived from the second-order polynomial strain potential energy measured by *in vivo* ultrasound. A hyperelastic material model is used to define the soft tissue portion of the model, as shown in Eq. 1:

$$U=\sum_{i+j=1}^{2}C_{ij}{\left({\bar{I}}_{1}-3\right)}^{i}{\left({\bar{I}}_{2}-3\right)}^{i}+\sum_{i=1}^{2}\frac{1}{D_{i}}{\left(J_{el}-1\right)}^{2i}$$ (1)

U is the strain energy per unit reference volume; C_ij_ and D_i_ are material parameters. J is the volume ratio; I_1_ and I_2_ are the 1st and 2nd deviator strain invariants. The superelastic material coefficients used for soft tissue are C_10_ = 0.08556, C_01_ = −0.0841, C_11_ = −0.02319, C_02_ = 0.00851, D_1_ = 3.65273, D_2_ = 0 (Lemmon et al., 1997). Material properties for each component are detailed in Table 1 (Siegler et al., 1988; Gefen, 2002; Cho et al., 2009; Gu et al., 2010; Brilakis et al., 2012; Gu et al., 2012). Cheung et al. (2004) simulated the effect of changing the stiffness of the PF on plantar pressure and the biomechanical interaction between bones and ligaments by changing the Young’s modulus of the PF. Therefore, we changed the Young’s modulus in the range of 135–405 MPa. Various values of the modulus are assigned to DTML to study the effect of FTA stiffness on load distribution. A Young’s modulus of 270 MPa was selected as the reference value to represent normal DTML stiffness (Gu et al., 2012), with the cross-sectional area of the fascia maintained constant across all simulation cases.

**TABLE 1:** Material properties of the components in the finite element model.

Component	Young’s modulus E (Mpa)	Poisson’s ratio ν	Size	Elements	Nodes	Reference
Bony Structures	7300	0.30	2 mm	253,217	709,301	Gefen (2002)
Soft tissue	—	—	3 mm	203,652	502,541	Cho et al. (2009)
Cartilage	1	0.40	0.5 mm	103,542	312,358	Gu et al. (2010)
Ligament	260	0.40	\	\	\	Siegler et al. (1988)
Planter fascia	350	0.40	1 mm	85,423	214,528	Brilakis et al. (2012)
DTML	270 (135–405)	0.40	1 mm	98,456	245,627	Gu et al. (2012)
Ground plate	17,000	0.10	3 mm	170	1360	Gu et al. (2010)

### 2.5 Boundary and loading conditions

**FIGURE 2:**
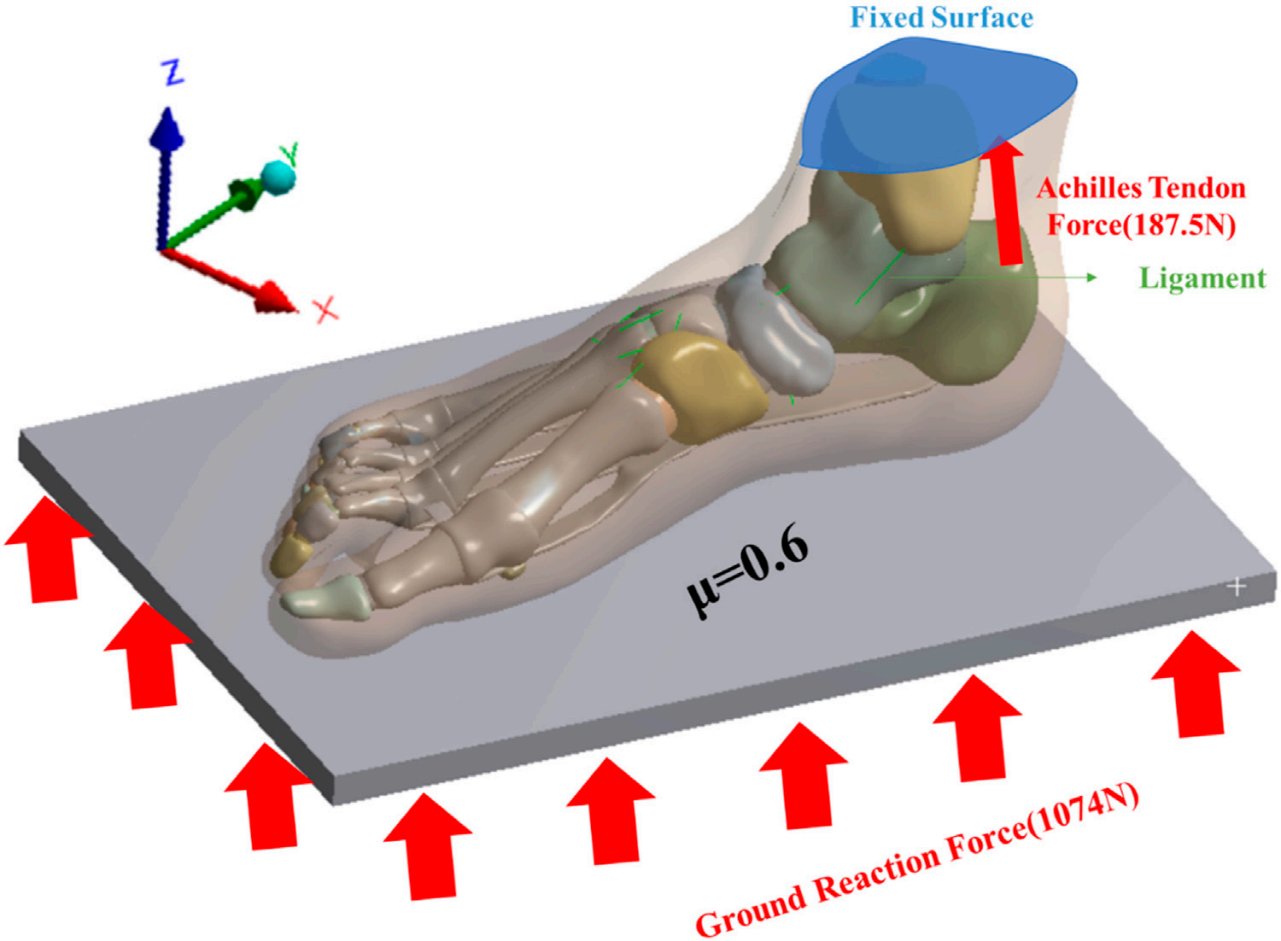
Loading and boundary conditions for FE analyses.

This study investigated the impact of FTA stiffness on the biomechanics of the foot and ankle complex during running. The AMTI force plate (Advance Mechanical Technology Inc., Watertown, NY, United States) was utilized to capture the force exerted by the subject’s right foot from ground contact to lift-off. Ground reaction force was recorded at a frequency of 1,000 Hz, with running speed determined by the subject’s self-selected pace. The number of experiments is three, and the interval between each experiment is 3 min. A flexible metal plate, capable of vertical movement only, was employed to simulate the ground (Kasiri-Bidhendi et al., 2015). The upper surface of the soft tissue, distal tibia, and distal fibula were fixed, as depicted in Figure 2. The average value of the second peak vertical ground reaction force of 1074N measured by the force plate is applied to the bottom of the metal plate as the ground reaction force of the FE analyze. Interaction between the foot and the plate was simulated as a contact surface with a friction coefficient of 0.6 (Yu et al., 2008). An equivalent force vector representing the Achilles tendon force was applied to the posterior aspect of the calcaneus. The magnitude of the Achilles tendon force was estimated as half of the reaction force (187.5 N) exerted by one foot when maintaining balance (Cheung et al., 2004).

### 2.6 Experimental validation

The model’s validity was assessed by comparing plantar pressure computed via FE analysis with plantar pressure measurements obtained using an Emed pressure plate (Novel, Munich, Germany), both conducted on the same subject. The subject was instructed to stand stationary on the Emed pressure plate for a duration of 5 s. Data collected during the middle 3 seconds were selected and averaged for analysis. This procedure ensured a representative assessment of plantar pressure distribution during static stance (El-Sallam et al., 2013).

## 3 Results

### 3.1 Model verification

**FIGURE 3:**
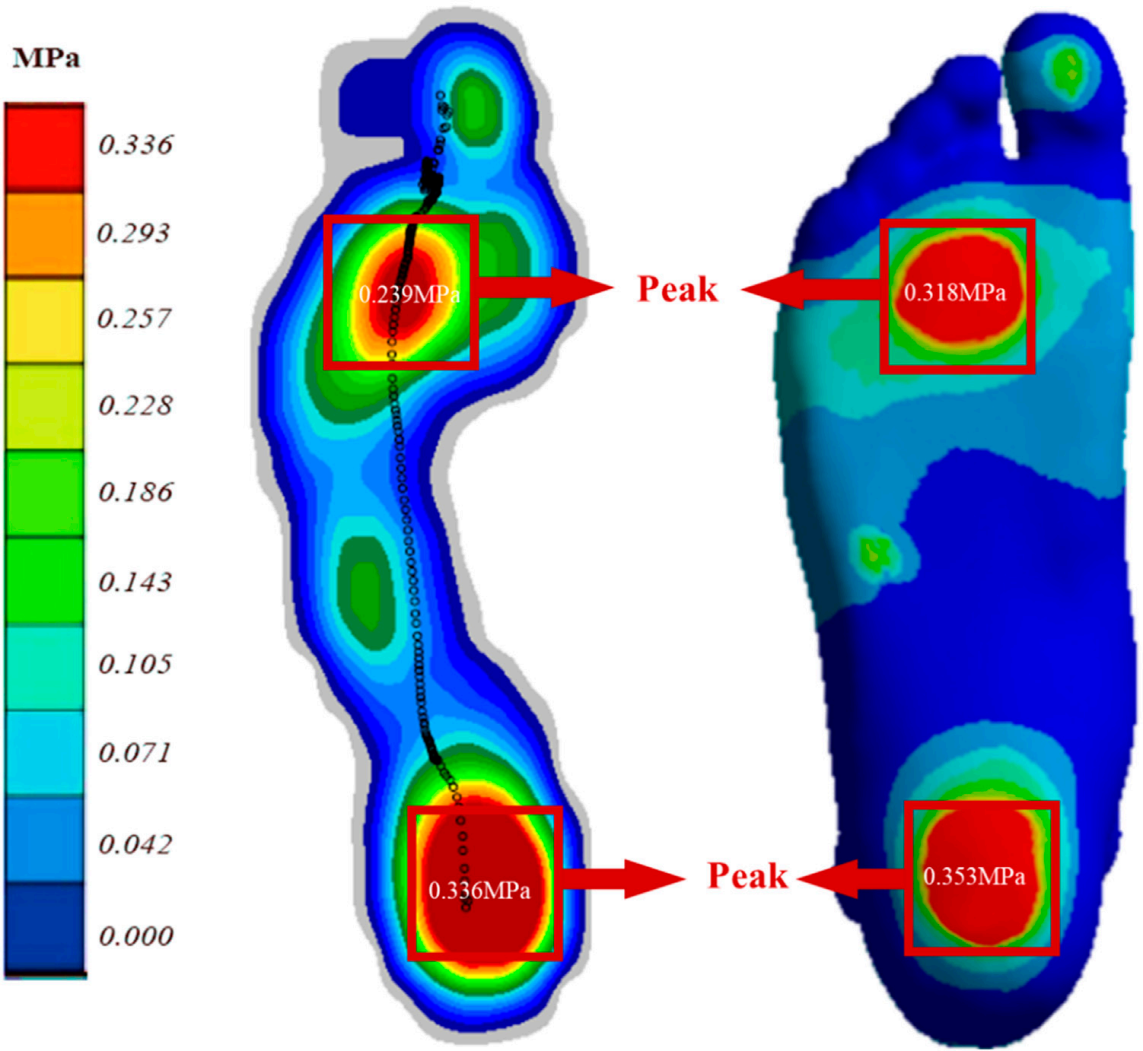
Comparison of FE predicted (right) and experimentally measured (left) peak pressure during balanced standing for model validation.

Figure 3 displays the plantar pressure distribution predicted by the Emed pressure plate and FE analysis while the subject maintained balance. The FE model utilized a DTML Young’s modulus (E) of 270 MPa as the reference value. Notably, the FE model demonstrates strong agreement with experimentally measured plantar pressure distribution and pressure values. Specifically, the simulated forefoot plantar peak pressure is 0.318 MPa, closely aligning with the measured value of 0.293 MPa, while the simulated and measured hindfoot plantar peak pressures are 0.353 MPa and 0.336 MPa, respectively.

### 3.2 Plantar pressure

**FIGURE 4:**
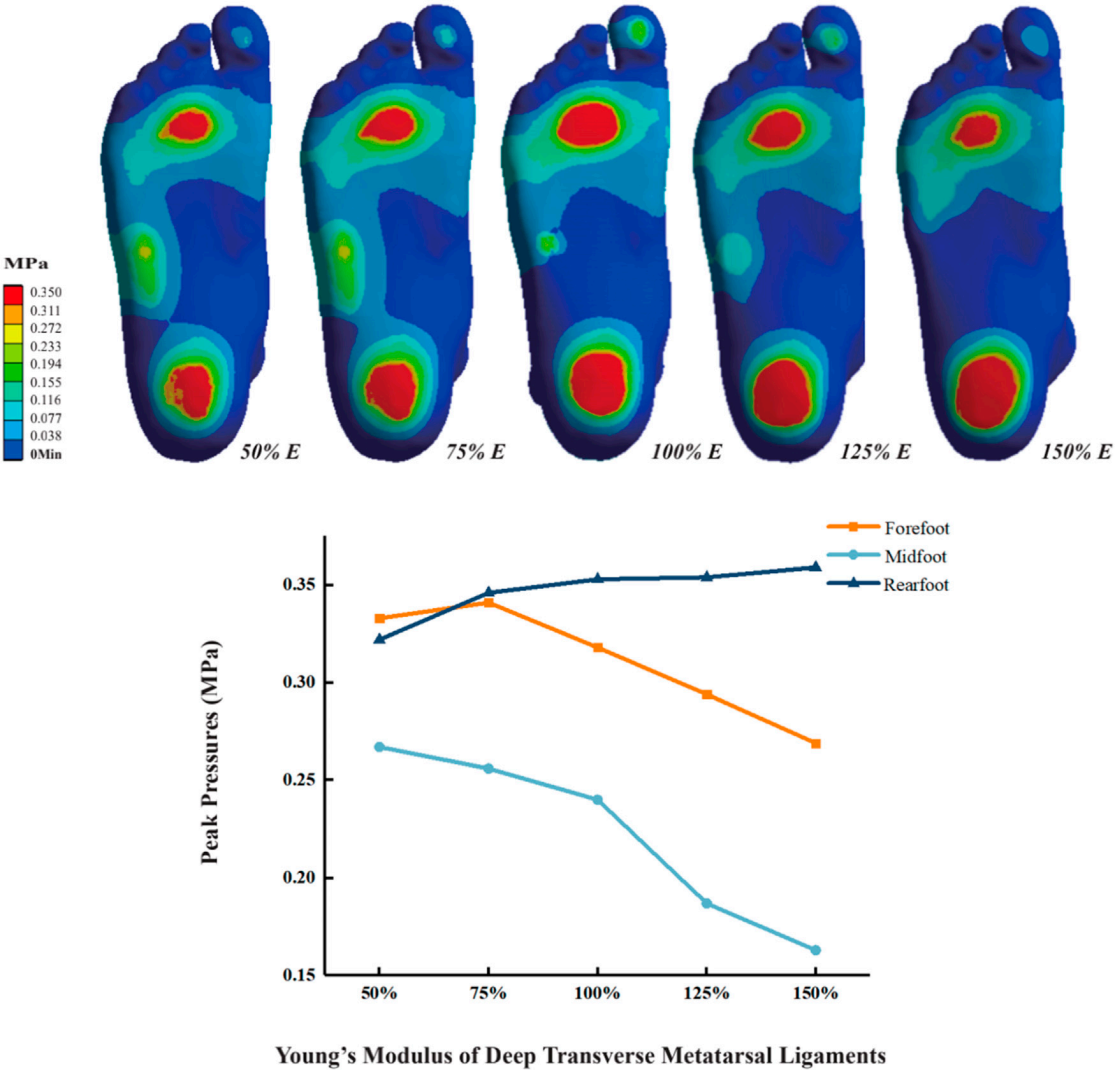
Effect of different DTML Young’s modulus on plantar peak pressure.

As the Young’s modulus of DTML increases, peak plantar pressure decreases, accompanied by pressure redistribution. From DTML Young’s modulus E = 135 MPa to E = 405 MPa, forefoot peak plantar pressure initially increases before exhibiting a decreasing trend (Figure 4). Compared to the reference value E = 270 MPa, when the Young’s modulus of DTML is reduced by 50%, the peak pressure of the forefoot increases by 4.7% (0.334 MPa), the peak pressure of the midfoot increases by 11.3% (0.267 MPa), and the peak pressure of the rearfoot decreases by 8.8% (0.322 MPa). When the Young’s modulus of DTML increases by 100%, the peak pressure of the forefoot and midfoot decreases by 19.2% (0.269 MPa) and 39% (0.163 MPa) respectively, while the peak pressure of the rearfoot increases by 11.4% (0.359 MPa). The overall foot plantar pressure is reduced by 14.2%.

### 3.3 PF strain

**FIGURE 5:**
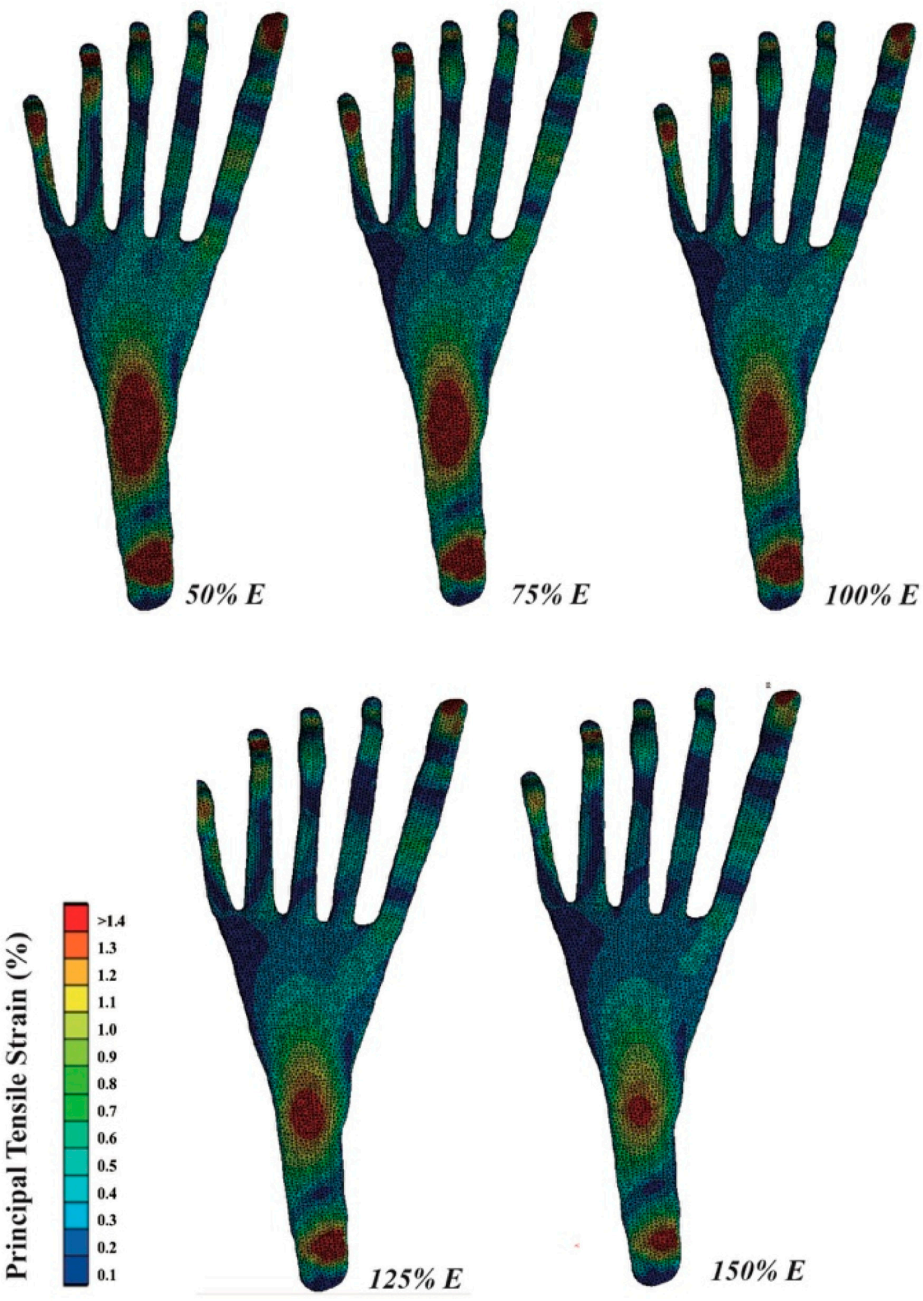
Graphic representation of plantar fascia strain distribution.

The tensile strain distribution of the PF, as simulated by FE, is depicted in Figure 5. Increasing the Young’s modulus of DTML effectively reduces the peak strain across various areas of the PF, particularly evident in the distal and middle segments. A 100% increase in DTML Young’s modulus correlates with a 38.5% reduction in the strain stretch of the PF.

### 3.4 Foot height and length

**FIGURE 6:**
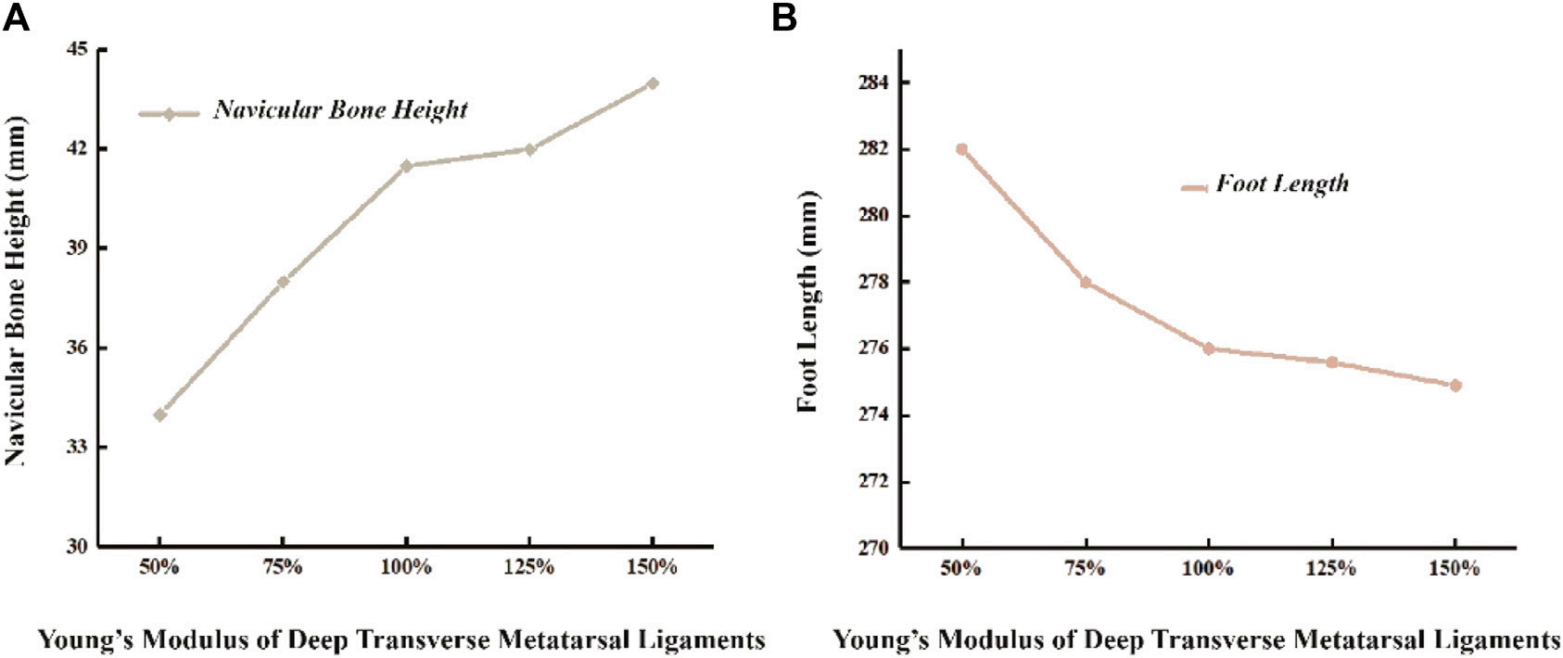
Effects of different DTML Young’s modulus on changes in **(A)** navicular bone height and **(B)** foot length.

In the unloaded simulated state, the height of the scaphoid measures 52.5 mm, with a foot length of 273 mm. During running, the scaphoid experiences a 21% reduction, measuring 41.5 mm. When the Young’s modulus of DTML decreases to 50% of the reference value E = 270 Mpa, the scaphoid drops to 34 mm. Conversely, under the condition of DTML Young’s modulus E = 405 Mpa, the scaphoid measures 44mm, representing an 8.5 mm reduction compared to the unloaded state (Figure 6A). Altering the Young’s modulus of DTML by ± 50% from the reference value results in a 0.4% decrease and a 2.1% increase in foot length, respectively (Figure 6B).

### 3.5 Metatarsal stress

**FIGURE 7:**
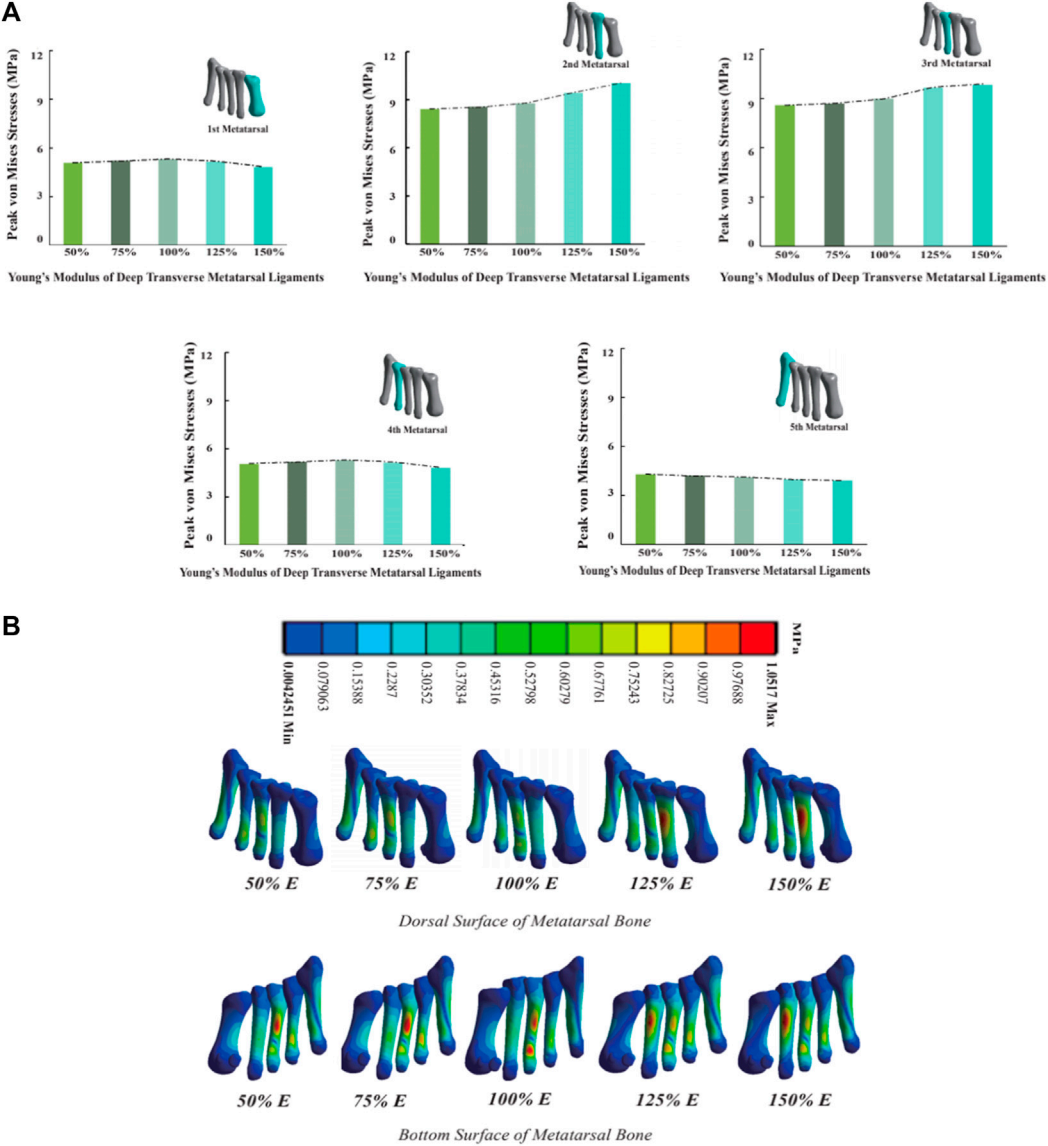
**(A)** Histogram and trend curve of the peak von Mises von Mises stress of the 1st-5th metatarsal bones; **(B)** Peak von Mises stress distribution cloud diagram of the 1st-5th metatarsal bones under the influence of different DTML Young’s modulus.

As the Young’s modulus of DTML increases from one-half the reference value, metatarsal von Mises stress generally escalates, except for M5 (Figure 7A). Comparatively, at 1.5 times the Young’s modulus reference value, the von Mises stress on the 2nd and 3rd metatarsals increases by 14.7% and 9.3%, respectively. Conversely, the von Mises stress on the 1st and 4th metatarsals decreases by 8.9% and 6.1%, respectively. The von Mises stress on the fifth metatarsal bone diminishes by 8.4% within the 100% change range of DTML Young’s modulus. With increasing DTML Young’s modulus, the von Mises stress distribution of the metatarsals becomes more concentrated. The von Mises stress on the 1st, 4th, and 5th metatarsals tends to shift towards the 2nd and 3rd metatarsals. Furthermore, the von Mises stress center also shifts vertically, transitioning from the base of the 3rd metatarsal to the posterior aspect of the 2nd (Figure 7B).

## 4 Discussion

Despite the acknowledged significance of the FTA in contributing to foot stiffness (Yawar et al., 2017; Venkadesan et al., 2020), detailed insights into the precise impact of FTA stiffness on internal foot structures remain limited. To comprehensively investigate the biomechanical response of the FTA within the context of internal foot structures, this study developed a subject-specific three-dimensional FE model of the foot-ankle complex and systematically assessed the quantitative influence of FTA stiffness on plantar load-bearing characteristics and internal foot structural parameters. Experimental findings indicate that progressive increases in FTA stiffness consistently reduce peak plantar pressure, as well as tension and strain levels within the PF, and mitigating scaphoid subsidence. Notably, variations are observed in the effects of FTA stiffness on the von Mises stress distribution across the five metatarsal bones. These nuanced biomechanical responses underscore the complexity of foot mechanics and highlight the need for further investigation to elucidate the precise interplay between FTA stiffness and internal foot structures.

As the Young’s modulus of the DTML increased from 50% to 150% of the reference value (E = 270 MPa), a notable decrease of 14.2% in peak plantar pressure was observed. This reduction primarily manifested as a decrease in peak pressure in the midfoot and forefoot regions. However, a rising trend in forefoot peak pressure was observed as the DTML Young’s modulus increased from 50% to 75% of the reference value. This phenomenon may be attributed to the increased stiffness of the FTA, which impedes midfoot sinking and redistributes pressure towards the front and rear feet. Despite the increase in the Young’s modulus of the DTML during this period, it proved insufficient to fully convert all increments of forefoot plantar pressure into elastic potential energy stored within the ligament. With further escalation in the lateral stiffness of DTML, the mechanical coupling between the foot’s sagittal plane bending and metatarsal head opening becomes more pronounced. This coupling facilitates the conversion of a greater proportion of ground reaction forces into the elastic force exerted by DTML, consequently reducing peak plantar pressure. Viewed from this perspective, the mechanical interplay between foot dorsiflexion and metatarsal head opening, along with increased FTA lateral stiffness, effectively reduces plantar pressure. Conversely, elevated plantar pressure, particularly in the forefoot, may heighten the risk of injury during movement (Wilzman et al., 2022).

The PF represents a crucial passive stabilizer in maintaining midfoot stiffness. In this study, a three-dimensional model of the PF was constructed to investigate its association with the FTA. Experimental findings revealed that a reduction in the Young’s modulus of the DTML resulted in increased peak strain within the PF. The deep PF inserts into the interosseous fascia, deep transverse plantar ligament, metatarsophalangeal joint plantar ligament, periosteum, and fibrous sheath at the base of each proximal phalanx, forming a sheath surrounding the flexor tendons (Davies, 2005). A decrease in the stiffness of the DTML disrupts the stability of the metatarsophalangeal joint, causing the metatarsals to expand along the Le Lie`vre metatarsal parabola. Consequently, the distal end of the PF undergoes increased passive stretching. This abnormal stretching of the distal end of the PF may contribute to forefoot pain associated with FTA dysfunction, such as hallux valgus (Nakai et al., 2019). Additionally, reduced stiffness across the foot, stemming from decreased forefoot lateral stiffness, elevates strain in the mid PF and heel, potentially exacerbating PF strain and predisposing to conditions like plantar fasciitis (Buchbinder, 2004; Irving et al., 2006; Wearing et al., 2006). Conversely, augmenting the stiffness of the FTA can effectively alleviate peak strain on the PF, thereby mitigating the risk of PF injury attributable to excessive fatigue.

Research findings indicate that augmenting the Young’s modulus of the DTML effectively prevents scaphoid collapse. A 100% increase in the Young’s modulus of DTML from half the reference value (E = 270 MPa) results in a 54.1% reduction in scaphoid subsidence and a 33.1% increase in midfoot stiffness. Through a combination of experiments and FE simulations, this study presents, for the first time in a foot model, the crucial role of the FTA in maintaining arch shape and enhancing foot stiffness. Previous studies primarily inferred the contribution of the TA to foot stiffness through mechanical models and mathematical methods, estimating contributions ranging from 40% to 50% (Yawar et al., 2017; Venkadesan et al., 2020). The contribution of the FTA to foot stiffness fundamentally differs from that of the PF. Whether through the bow-string configuration or the windlass mechanism, both aim to increase tension of the PF to resist flattening of the bony arch under gravity. The inherent stiffness of the foot’s arch structure, mediated by the joint capsule, key ligaments, and muscles, is augmented more directly by the FTA through alterations in lateral arch curvature, metatarsal bone expansion, and dorsalis curvature coupling. While medial arch support insoles have historically been favored for flat-footed patients and proven effective in symptom relief (Su et al., 2017; Wahmkow et al., 2017; Peng et al., 2021), this method may inadvertently increase pressure on the medial midfoot region, potentially leading to long-term discomfort. Additionally, excessive arch support from foot orthotics can impose undue von Mises stress on the foot-ankle complex’s articular cartilage and ligaments (Su et al., 2017). The research suggests that enhancing arch stiffness through adjustments in FTA curvature and lateral expansion may offer a novel therapeutic avenue for treating flat feet, potentially mitigating the need for excessive arch support and minimizing associated risks of discomfort and structural strain in the foot-ankle complex.

As the Young’s modulus of the DTML increases, von Mises stress on the first, second, and third metatarsals also increases, with a concentration of von Mises stress towards the center. Despite the reduction in peak forefoot pressure, the second and third metatarsal bones still experience elevated peak von Mises stress levels, which may partly explain why most metatarsal von Mises stress fractures occur in these regions (Sullivan et al., 1984). It is noteworthy that when the DTML Young’s modulus reaches 150% of the reference value (E = 270 MPa), peak metatarsal von Mises stress shifts from the base of the third metatarsal to the dorsal side of the second metatarsal. This phenomenon of von Mises stress transfer may be attributed to alterations in the forces acting on the metatarsal bone. With low DTML stiffness, the maintenance of the FTA shape is compromised, leading to shear forces at the base of the metatarsal heads under the influence of gravity and ground reaction forces, thereby concentrating von Mises stress at the base of the third metatarsal bone. As the stiffness of DTML increases, the second metatarsal rises to become the apex of the FTA. Consequently, shear forces diminish, and the dorsal aspect of the metatarsal experiences downward pressure, resulting in a concentrated peak von Mises stress at the proximal end of the second metatarsal. In contrast, the peak von Mises stress on the fourth and fifth metatarsals decreases proportionally as pressure diminishes.

Further experimental research is necessary to ascertain whether alterations in von Mises stress due to DTML stiffness have a discernible impact on the risk of injury. This will help elucidate the biomechanical implications of foot structure and function under varying ligamentous stiffness conditions, contributing to a deeper understanding of foot mechanics and injury prevention strategies.

It is important to acknowledge potential limitations inherent in this study. Firstly, the reliance on data from a single individual for all simulations may raise concerns regarding the generalizability of the results to broader populations. The use of a single subject limits the ability to capture variations in foot biomechanics across different individuals (Wong et al., 2021). Follow-up studies should consider multiple human sample models for study (Zhan et al., 2024). Secondly, while this study examined internal effects through intra-test differences, it did not assess external effects, which may limit the generalizability of the research conclusions (Chen et al., 2020). In terms of materials, except the wrapped soft tissue, all materials are isotropic linear elastic materials. Bone is divided into cortical bone and cancellous bone. If a bone is defined as a linear elastic material, the stress value of the bone will increase, which requires simplifying some secondary tissues and structures of complex organisms, which cannot be completely accurate. In addition, due to the use of the FE method, the results are based on some assumptions, which may be reflected as potential limitations (Malakoutikhah et al., 2022). Therefore, improving the geometric similarity and accuracy of the FE model is an important direction of biomechanical FE analysis.

## 5 Conclusion

To our knowledge, this study represents the first attempt to investigate the influence of the transverse arch on midfoot stiffness by quantifying its impact on internal load-bearing characteristics of the foot. Through experimental validation, we have confirmed that augmenting the stiffness of the transverse arch effectively enhances the overall stiffness of the midfoot. As a result, we recommend considering methods aimed at preserving the shape and increasing the stiffness of the forefoot transverse arch when addressing symptoms associated with medial longitudinal arch collapse in the foot. Furthermore, preserving the shape and curvature of the TA could serve as a strategy in the design of running shoes to enhance the stiffness of the foot during running.

## Data Availability

The original contributions presented in the study are included in the article/Supplementary Material, further inquiries can be directed to the corresponding author.
